# “Working in the content factory”: musicians’ social media use and mental health as seen through the lens of a transdiagnostic cognitive behavioural conceptualisation

**DOI:** 10.3389/fpsyg.2025.1542407

**Published:** 2025-08-01

**Authors:** George Musgrave, Daniel Carney, Emma Silver, Marc S. Tibber

**Affiliations:** ^1^Institute for Creative and Cultural Entrepreneurship, Goldsmiths (University of London), London, United Kingdom; ^2^Research Department of Clinical, Educational and Health Psychology, University College London, London, United Kingdom

**Keywords:** musicians, social media, social networking sites, mental health, wellbeing

## Abstract

Research shows that musicians are an at-risk occupational group for mental health difficulties and suicidality. Further, social media has become central to working musicians’ lives, and there is a growing concern that social media may be linked to the increasing prevalence of mental health difficulties within the general population. Despite this, few studies have explored the role of social media in musicians’ mental health and wellbeing, both in terms of benefits to harness, as well as harms to avoid. Drawing on a transdiagnostic cognitive behavioural conceptualisation of social media use and mental health links, this interdisciplinary qualitative article draws on semi-structured interviews with twelve musicians from across the United Kingdom building careers in genres of popular, i.e., non-classical, music. Findings from thematic analysis highlighted potential benefits and harms of social media engagement, e.g., opportunities for social connection, self-expression, networking, career building, and as a source of inspiration, as well as the possibility of social disconnection, harmful social comparisons, experiences of stigma, trolling and abuse, uncertainty around the nature of the algorithm, and a sense of needing to share more and more, with a risk that it starts to displace valued offline activities. We explore these findings through the lens of a transdiagnostic conceptualisation, and highlight clinical implications aimed at supporting musicians to use social media in ways that support their wellbeing.

## 1 Introduction

A key facet of the digitalisation of music distribution and consumption has been the rise of social media (SM)^1^ as an artist marketing and communication tool. Optimistic music industry discourses around SM have emphasised the new ‘participatory culture’ (Jenkins, 2006) enabled by Web 2.0, through which relationships between artists and audiences are reconfigured (Beer, 2008). Musicians, so this argument goes, can now use SM to reach new fans directly (Watson et al., 2023), engaging with them in more intense, direct, and rewarding ways (Bennett, 2016), and supplanting traditional industry intermediaries (Simon, 2019). Consequently, building and maintaining an online presence is increasingly seen as a central part of a professional musician’s job (Gee and Yeow, 2021), a key feature of modern ‘*music entrepreneurship’* (Dumbreck and McPherson, 2016), and even the ‘cornerstone’ of a musician’s career (Kusek, 2014).

Existing literature suggests career musicians—by which we mean those who pursue the writing, composing, recording and/or performing of music as an occupation—have high levels of occupational stress (King et al., 2024), and are at increased risk of suicidality (Kenny and Asher, 2016; Musgrave and Lamis, 2025) and early mortality (Bellis et al., 2007, 2012) relative to non-musicians. Likewise, heightened incidences of mental health conditions such as anxiety and depression have been observed among this population relative to the general public (Kegelaers et al., 2022; Vaag et al., 2016) with some research adopting a psychosocial lens suggesting that the nature of musicians’ working conditions might be contributory factors, rooted in features such as financial precarity (Berg et al., 2022) and sexual discrimination (Conor et al., 2015); see review by Jepson et al. (2024). Against this backdrop, it is unclear to what extent the aforementioned push towards greater online presence plays a part in this occupational stress (or its alleviation).

While recent articles in the popular media have lamented the impact of SM usage on specific musicians’ mental health (e.g., Daly, 2020), few empirical studies have examined the role of SM in musicians’ mental health and wellbeing; see Woods and Davis (2024) on digital burnout and Lee (2024) on creative workers in the television industry, however. This lack of scholarly attention is particularly notable given the growing body of literature highlighting potential negative and positive impacts of SM use on mental health and wellbeing in the general population, albeit with methodological and theoretical limitations, which (for example) often preclude conclusions about underlying directions of causality, e.g., Popat and Tarrant (2023).

Much early research into the potential impact of SM in the general population adopted a deterministic ‘concern-centric’ perspective assuming that SM is inherently harmful, documenting impacts on a range of outcomes including: psychological functioning (Sampasa-Kanyinga and Lewis, 2015), self-esteem (Fiovaranti et al., 2012), depression (Giota and Kleftaras, 2013; Wright et al., 2013), anxiety (Xiuqin et al., 2010), and loneliness (Song et al., 2014), particularly in adolescents, e.g., Odgers and Jensen (2020). This focus on harms also extends to the development of theory within the field, and while numerous models of ‘problematic’ technology use exist, e.g., models of SM ‘addiction’ and related (Sun and Zhang, 2021), these do not typically accommodate parallel *benefits* of the technology which are also well documented in the literature (e.g., Craig et al., 2021; Foster, 2015). While such models may be useful in certain clinical contexts, we would argue that without a consideration of the potential *benefits* of engagement alongside the potential costs, our understanding of both the causes and consequences of SM use, as well as ways to support healthy engagement, will be limited.

In this context there have been calls recently for a more nuanced perspective of SM (Hesmondhalgh and Kaye, 2025), and a growing recognition of the potential benefits *alongside* the harms of engagement, as well as the complex interacting nature of individual and technological factors in determining these (Tibber and Silver, 2022). For example, there is evidence that certain kinds of musicians might suffer from high levels of emotional sensitivity and instability, fearfulness (Kemp, 1981, 1996), and anxiety (Musgrave et al., 2025), and that anxious SM users of this kind may be more likely to engage with SM in order to manage these emotions (Primack and Escobar-Viera, 2017), and will often turn to the online world to seek attention, support, and/or acceptance/belonging (Vannucci et al., 2017). Whether these needs are subsequently met online, however, is clearly not guaranteed.

With respect to the potential harms of SM engagement for musicians, examples include trolling or other forms of abuse (Gross and Musgrave, 2017), with specific vulnerabilities for women (Choi, 2016). Likewise, the expectation that musicians open up their lives to fans online, acting as ‘entrepreneurial attention seekers’ who must be constantly accessible (Tessler and Flynn, 2016) and offer unique and intimate moments to fans (Baym, 2015) may present relational challenges. Such context collapse (Baym, 2012) may also lead to burnout or fatigue (Woods and Davis, 2024). Likewise, musicians may be impacted negatively by online ‘upward’ social comparison, i.e., comparisons made towards someone deemed better off than oneself on some dimension (Gross and Musgrave, 2020), with potential knock-on effects on mental health and wellbeing observed from this phenomenon (Tandoc et al., 2015), particularly in the context of an increasingly competitive online marketplace (Ng and Gamble, 2024).

With respect to potential benefits for musicians, SM offers up-and-coming musicians the opportunity to develop networks and relationships online, which are seen as essential for their careers (Coulson, 2012; Musgrave, 2023b), in a way that was previously impossible for those outside major cities, and which is often welcome by musicians (Haynes and Marshall, 2018). Similarly, Baym (2012) highlights the sense of community and social support that can be provided by musicians’ online networks, and studies have shown social support to be positively related to musicians’ wellbeing (Liljeholm Johansson and Theorell, 2003) as well as negatively related to their psychological distress (Aalberg et al., 2019). Other potential benefits include feelings of empowerment from political activism and collective action, as explored among Black musicians and fans in the work of Dennis (2016), or Cuban musicians by Weener and van Noorloos (2022), for example.

While these studies indicate a growing interest in the role of SM in career-musicians’ lives, there is a relative dearth of research exploring the complex and nuanced ways in which SM use interacts with mental health and wellbeing in this population. Further, as noted, the existing research that does exist often lacks theoretical integration outside of clinical and/or pathological patterns of use, without which, it has been argued, a field cannot progress (Orben, 2018). Given the increased vulnerability of this population to mental health difficulties, and growing ubiquity of SM use, we believe this is a critical area for research with potentially important implications for the wellbeing of artists.

Against this backdrop, this study reports on in-depth interviews conducted with UK-based musicians working in genres of popular music, i.e., non-classical and non-jazz music genres. Using a qualitative approach, this study sought to compliment and build upon existing quantitative work (Keles et al., 2019), providing a rich and contextual perspective of musicians’ experiences of SM use. This interdisciplinary study brought together specialists working in the fields of cultural sociology and psychology, and clinical psychologists working professionally with musicians, to empirically examine the complex roles that SM plays in musicians’ mental health and wellbeing.

In order to aid development of the interview schedule and analysis codes, as well as interpretation of the findings, we drew upon Tibber and Silver’s (2022) transdiagnostic cognitive behavioural conceptualisation of SM use (herein TCBC). This SM-specific TCBC was explicitly developed to address the aforementioned limitations of theory within the field and conceptualises both the positive *and* negative roles of SM in mental health and wellbeing. The overarching aims of the TCBC’s development were to: (i) provide a framework to stimulate further research into SM and mental health/wellbeing links, and (ii) help mental health professionals to formulate the role of SM in their work, in the hope of ultimately (iii) developing resources and interventions to help people flourish on- and off-line. For full details of the conceptualisation we would direct the reader to the original paper. However, in brief, Tibber and Silver’s (2022) TCBC seeks to understand how individual and technological factors interact to differentially dispose the user towards potential harms and benefits of use, and further, how these processes relate to common mental health difficulties (namely anxiety and depression). Crucially, the conceptualisation proposes that the benefits of SM engagement are more likely to be accrued when use is purposeful/intentional (i.e., mindful), underpinned by motivations aligned with the user’s values, and further, when online behaviour is aligned with these. The TCBC suggests that under these conditions core needs, particularly those relating to autonomy, relatedness and competence are more likely to be met (Ryan and Deci, 2017; Orben et al., 2020); this is critical, since satisfaction of such core needs have been robustly linked to a range of positive mental health and wellbeing outcomes (Sheldon et al., 2004; Bradshaw, 2023). Finally, the TCBC proposes that SM is neither *inherently* helpful or harmful, but a tool, which can both support and hinder satisfaction of such core needs (sometimes in parallel). Nonetheless, the model recognises that certain features and affordances of platform design, embedded as they are for musicians within an ‘attention economy’ (see Morrow, 2018), may pull the user towards greater levels of engagement, and less mindful engagement, which must be actively resisted if these tendencies are to be overcome.

## 2 Method

### 2.1 Participants

Ethical approval was granted by the Goldsmiths (University of London) Research Ethics and Integrity Sub-Committee (Reference 1780, 7th November 2023). Participants were recruited via an open call on SM and through snowball sampling to ensure diversity of participants. Inclusion criteria included being 18 years or over, being based in the UK, and pursuing a career in the music industry (as opposed to music as a hobby or leisure activity). Within this remit we sought diversity with respect to age, gender, ethnicity, and geographical location, as well as musical genre, professional musical identity (e.g., DJs, singer songwriters, guitarists), career stage and status (see Table 1). Of the twelve participants interviewed (6 male, and 6 female), four (33%) were signed with major (or culturally significant independent) record labels and/or management companies and had large reserves of (sub)cultural capital (Thornton, 1995), four (33%) might be thought of as mid-career musicians developing their online profiles and beginning to acquire a reputation and status, while two (16.5%) were early career artists, and two (16.5%) did not place music as a career as centrally in their lives as the other participants. Six were based in London—reflecting the dominance of London in musicians’ career ambitions (Watson, 2008)—while the remaining six came from Birmingham, Essex, Bournemouth, Manchester and two from Norfolk. Nine (75%) were White, two (16.5%) were Black, and one (8.3%) was Black/mixed race. Ages ranged from 25 to 45 years, with a median age of 31.6. Musicians came from a range of genres. A systematic review (Hennink and Kaiser, 2022) suggests the saturation point for qualitative research typically occurs between nine and seventeen interviews, notably where study populations are relatively homogenous and objectives narrowly defined. It was ascertained that saturation had been achieved after the twelfth interview.

**Table 1:** Demographics and other key information of participants.

Participant	Genre	Age	Gender	Ethnicity	Musical identity
1	Electro	41	Male	White	Producer, artist
2	Pop/rock	29	Male	White	Solo singer songwriter
3	Acoustic, folk	25	Female	White	Solo singer songwriter
4	EDM	30	Female	White	Producer, DJ
5	Various UK underground genres	27	Female	Black/mixed	DJ
6	Alt-rock, post-punk	45	Female	White	Solo singer songwriter
7	Electro pop	27	Female	White	Singer songwriter
8	Electro pop	27	Male	White	Singer songwriter
9	House	37	Female	White	Producer, DJ
10	Punk	27	Male	White	Guitarist, lead vocalist
11	Black Music	Under 40	Male	Black	DJ, producer and artist manager
12	Hip hop/rap, electronica, experimental, African	27	Male	Black	Producer, live performance artist

Interview questions were constructed in accordance with conceptual categories provided by the TCBC (Tibber and Silver, 2022) as well as themes emerging from the extant literature and clinical experience of working with musicians. Specifically, participants were asked about: (1) their SM use in general (e.g., platforms used, time spent online, etc.), (2) their motivations for engagement, (3) the nature of their online behaviours, (4) commonalities and differences in experiences across different SM platforms, (5) commonalities and differences in participants’ online and offline identities, (6) the role of fans/audiences, (7) their mode of engagement (active versus passive; intentional/purposeful versus automatic/habitual), (8) the consequences of use, i.e., costs and benefits, and (9) any desired changes participants identified with respect to their SM use.^2^

### 2.2 Procedure and data analysis

All participants were contacted by the lead author and a participant information sheet and consent sheet provided. Participants based in London were interviewed in-person at a location of their choosing; those elsewhere in the UK were interviewed remotely (Teams or Zoom). No reimbursement or payment was offered. Interviews were recorded and transcribed by the lead author (GM).

Interviews were thematically analysed (Clarke and Braun, 2018) in two stages. First, template analysis (Brooks et al., 2015) was undertaken by DC, with transcripts coded and marked up with respect to nine *a priori* categories (defined above). Four of these scripts were then additionally coded by two other clinical authors (ES and MST) to ensure consistency. These codes were then re-analysed by GM to find commonalities and differences in experiences across and between participants and to identify bottom-up themes to emerge from the interviews.

## 3 Findings

All twelve participants were highly active SM users, with the majority using it daily for several hours and across a range of social networking sites (SNS), though predominantly Instagram, TikTok and X (formerly Twitter), as well as Facebook (with only one mentioning LinkedIn). Five first-order themes were extracted from transcription coding: (i) connection, (ii) the role of ‘control’, (iii) the experience of comparison, (iv) making content, drawing boundaries, and finally (v) moderating factors, which could be broken down further into second and third order themes (see Table 2). These are discussed below.

**Table 2:** First, second and third order themes from coding.

First order themes	Second order themes	Third order themes
Connection	Building a career; feeling connected	Connecting with fans; connecting with industry; emotional connection as joy; social connections seen in metrics; translating metrics; SM as a CV
The role of ‘control’	External control; internal control	Passive scrolling and no control; the algorithm and control; desire for intentionality; compulsion to use SM for career progression
The experience of comparison	Upward comparison harming wellbeing; comparison as inspiration	
Making content, drawing boundaries	Identity; time	Public/private identity negotiation; SM impacting time/headspace; SM impacting ‘real life’ choices; exhaustion
Moderating factors	Career orientation and status; gender; race; attitude towards SM	

SM, social media; CV, curriculum vitae.

### 3.1 Connection: building a career and feeling connected

Mapping onto extant literature, participants’ responses to the questions “why do you use SM?” and “what do you use SM for?” made repeated reference to *connection*. However, as we will see below it was difficult to disentangle motivations to connect for the purpose of building a career, from motivations to connect with an audience (or for their music ‘to connect’) to feel connected, and in doing so satisfy core needs (we would suggest) related to acceptance and belonging through sharing their music.

All participants spoke of a motivation to forge and/or solidify connections within a highly network-dependent industry. SM was framed as a tool “to directly speak to brand managers and talent managers” (P5), and “to find leads” (P12)—a kind of network-building understood as “essential… it’s the way that A&Rs notice us, and managers notice us” (P7). Far more prominent for participants, however, was their desire to make their music heard by new and existing audiences against a backdrop of what Participant 9 called the “noise” of ambitious musicians on SM. For participants operating in less mainstream genres, where music as a career played a less central role in their working lives, and/or those with a smaller SM followings, this interactivity tended to play a ‘SM as advertising’ role, e.g., “a way of telling people we have gigs” (P10). For the majority, however, there was also a desire to forge deep and meaningful connections with listeners, and to tell their musical story and the “world of all the different things that I’m interested in… to keep the audience excited and connected to me: to my world” (P6). Through these connections, participants were wanting to be seen and heard, and feel that their music was connecting with others, in an abundant online marketplace where this was understood as both incredibly hard to find and hugely meaningful.

Two metrics of connection were identified, both of which oscillated around the idea of validation. The first was less tangible, but more intimate and qualitative, e.g., memories of times when audiences communicated with them personally and appreciated their music: “someone saying I love your song matters… [It] gives you the mental juice to keep going… Emotional connection…[is] the ‘glue’” (P1). This kind of emotional bond and sense of connection with an audience was one of the principal ways in which SM felt good to interviewees: “I’d be lying if I said that we weren’t over the moon when we sat in the car after [a gig] just looking for all the mentions of us [on Instagram]” (P10).

There were also quantitative markers of connection, e.g., ‘likes’, ‘shares’, and growth in the number of followers. However, these metrics of connection were generally deemed as less important and meaningful, e.g., “it’s not what we focus on” (P7). Nonetheless, this kind of engagement was also framed as being emotionally beneficial in some circumstances: “When we see something that goes well in those classic metrics of engagement, followers, comments, just all of that, I think it’s super positive” (P7). Perhaps most bluntly, Participant 1 said: “That’s the crack. That’s the drug: validation.”

The role of metrics in participants’ SM use also spoke to a symbolic function of this connection. Metrics such as ‘likes’, ‘shares’ and numbers of followers were often used as numerical representations of the strength and magnitude of the connection between a musician and their audience, acting therefore as symbols of status and cache, and a digital placeholder of achievements—akin to a curriculum vitae (CV). Ultimately, it was hoped that this could be converted via an imprecise process of metric translation into a variety of career-related opportunities and achievements, e.g., “bums on seats” (P6) at gigs, a greater number of streams on digital service providers (DSPs) such as Spotify, stronger relationships with music industry professionals, and, in the final and most hopeful instance, money, i.e., what Musgrave (2017) refers to as ‘secondary transubstantiation’ (turning prestige into income). For example, when asked why she used SM, Participant 4 stated: “To get fans and followers…to build myself as an artist and get my music heard more which then results in more Spotify streams… which is then going to lead to more ticket sales for me.” Her metrics being high were a key part of this, as she went on to say: “[I can] use those [SM] stats to send to radio or DSPs.” These metrics then become the symbolic mechanism of acquired (sub)cultural capital through which Spotify might add them to an editorially curated playlist, journalists might write about them, and venues might book them to play bigger shows; in essence, that the institutions of the music industry might take them seriously and their career would grow and develop.

However, it was often difficult for participants to be precise about how success and increased metrics in one domain, e.g., ‘likes’ or followers, connected to success in other domains, e.g., streams or money. Participant 1 called this a process of ‘network value’: “Some of the things you get nothing from but they bring value in other places…[and] all these pieces feed… [in a] messy network… So like when my streaming does well, it does not actually make me lots of money, but it looks good, right?. It’s kudos. ‘Followers’ is a metric of: this person’s serious… [It’s] a stamp of approval. That this person is generating interest on their own…It’s a signal.” How this signal *worked* was often less clear, however. This uncertainty over how practices of metric conversion took place, and the opaqueness vis-à-vis how to specifically identity the effectiveness of a musicians’ SM use is reflected in the fact that almost all participants stopped, paused, reflected and genuinely considered how to answer the question: ‘How do you know your SM use is working, or is successful?’

### 3.2 The role of ‘control’

Perhaps the most fascinating moment in every interview was when participants were asked the question: ‘When you engage with SM to what extent do you feel in control?’. The phrasing of this question was deliberately ambiguous to allow participants to interpret the meaning of the question, which they tended to do so in two ways, i.e., in terms of *external* and *internal* control.

With respect to *external control*, many participants perceived their SM use to be linked to how ‘in control’ they felt with respect to their career. On the one hand, SM had (as noted above) often afforded them the chance to forge and develop powerful, and at times intimate, relationships with listeners, which often went beyond parasociality and became sources of meaningful connectivity. At the same time, there was an acute understanding that this was occurring within a digitalised sphere over which their hard work and investment strategies (principally the investment of time and their creative efforts) were to an extent at the mercy of the platforms on which their work was circulating—Instagram, TikTok and X/Twitter. In other words, the quantification of career musicianship and its ultimate realisation in processes of metric conversion—a strategic and symbolic rationale for SM use—was subject to profound uncertainty as a central unknowable exercised a large influence over their career: the algorithm.

Participants would speak of pouring hours and hours, even years, of work into building their artistic careers on SM, and creating engaging content that (they hoped) would perform well online, but that this was often dependent on how well (or not) the algorithm would ‘push’ their content to listeners. This seemed to be particularly the case on TikTok. It was striking also how often the algorithm was often reified and even anthropomorphised as a figure looming large over participants’ creative lives within a language of randomness and uncertainty, e.g., “playing the lottery of the algorithm” (P11), “like this kind of mystical shaman on the hill” (P2), or “now the gatekeeper is an algorithm” (P8). Participant 1 phrased this as: “With the algorithm… people are just, like, ‘put out a song every three weeks [because] you don't know what’s good! Just keep putting out music and eventually something will hit the algorithm’… None of us know what the fuck we’re talking about.” This lack of certainty seemed to be mirrored at different levels and professions within the industry: “Management and all the old heads in the industry speak in banal aphorisms about the effect that social media has on the careers of artists without really having any clue what they’re talking about” (P2).

Critically, these algorithms were seen as responsible for the very visibility and connectivity with audiences that they were seeking to achieve (as per Woods and Davis, 2024). In other words, there was a sense in which participants were not in control over their work, its impact, and by extension their lives and careers. Participant 2 for example spoke of how writing a song that he felt proud of was a “priceless” thing that “truly matters” in life, but being frustrated that writing great music was “not a guarantee of anything and it feels massively like just fishing in the dark.” He went on to say: “it feels really weird to just distil the whole thing of music… down to a sense of rolling the dice, like just praying that an algorithm picks you up… That’s a bit of a mentally alienating thing” (P2). This sentiment was echoed again and again by many participants.

It appeared that the majority of participants were “playing the lottery of the algorithm” (P11), often encountering feelings of despondency when they lost, but simultaneously hooked by the ‘crack’ of the validation on the rare occasions that they won. In general, and as expressed by Participant 11, it appeared that when SM was being used as advertising it was negatively impacting participants’ wellbeing, but when it had a more creative orientation—and therefore perhaps more deeply connected to values such as connection, creativity and self-expression—it appeared to be more beneficial.

Alongside this broader, more fundamental (even existential) understanding of the relationship between the term ‘control’ and SM, participants also referred to a perceived lack of (*internal*) control over their own SM use. This concerned the fact that despite logging on to SM with clear intentions to post and engage, this often led to extended periods of passive scrolling, which was commonly described in the language of addiction and in terms of a need to take active steps to try and distance themselves from, e.g., “I’m getting better at recognising the control it has over me… I will put my phone upstairs. It has to just be like away… physically a bit removed from me” (P1); “[you need] the discipline of removing yourself from it when you need to for your own mental health and I think like that’s just something I need to work on personally” (P4), and “I’ve tried to make [the apps] harder to get into and also have the time limit thing on my phone … I’m trying to be a lot more intentional with it” (P9). While not all participants felt this way, being able to control their use was something many had explicitly worked on, whereby intentionality was a strategic attempt to overcome this drag towards mindless use. For example, Participant 5 had been in therapy and had discussed the role of SM in her life during therapy sessions, and in response had adopted firm boundaries around her use. However, this was relatively unusual and struggling to regulate usage was far more common.

To an extent, the pervasive and central role SM played in many participants’ lives is not unlike that expressed by non-musicians in the existing research, whereby the features and affordances of the technology are designed to maximise use (i.e., keep them ‘hooked’) and minimise deeper cognitive engagement (Tibber and Silver, 2022). However, there was a crucial difference in the lives of the musicians we spoke to, which was that they felt professionally *compelled* to be active online, with some of this compulsion emanating internally from their own ambition [“A big part of [why] I do it is because I *have* to… I have to do it because it’s such an important part of being a musician” (P3)], but sometimes coming from others in their professional network, e.g., “My management, it’s not like they put a gun to my head and say like, ‘You have to post.’ But they very much like imply that I have to post” (P2). This in itself fed into a perceived lack of control, i.e., that participants *had* to be present online given that the ‘gods of TikTok and the algorithm’ (P7) might pick you at any moment; participants felt they needed to stay perpetually engaged and “stay visible…to try and exist in the public eye” (P2) so that audiences do not “forget that I exist” (P9) in an online marketplace which was ferociously competitive. For some this came with profoundly negative impacts on their wellbeing: “It feels so close, like we’re just about to do it… success is in front of your face all the time” (P1), but it was not always clear how (and if) they could reach out and touch it.

### 3.3 The experience of comparison

All participants (bar one) spoke about social comparisons in response to the question: ‘Do the ways in which you use and experience social media affect your mood and/or mental health?’. Consistent with previous literature, negative impacts, particularly on self-esteem were driven by upward social comparisons (see Feinstein et al., 2013, for example) particularly comparing oneself to other musicians. For example, Participant 5 described comparisons as “the thief of joy… [My anxiety] just all falls under the umbrella of comparison… The constant bombardment of achievement”, and Participant 6 phrased this as “it has negatively affected my mental health just in the sense of every day I look and see what everyone else is doing and perceive that everyone’s doing better than me…It’s like waking up and punching yourself in the face”.

It is important to note that for some participants this kind of musician-to-musician comparison could also act as a source of motivating inspiration, which has sometimes been referred to in the literature as ‘benign envy’ (Tibber et al., 2024). This could be uplifting and a source of encouragement: “I’m always like looking for new trends. I’m saving posts to see what other people are doing like ‘oh that’s working, like, you know it’s gone viral, it’s popped off’, so I’ll copy that” (P4), and “I really do get inspired by tapping into socials at times where I feel like I am in a good place to do that” (P5). However, this was less common than the negative experiences of social comparisons.

In many respects, as suggested, participants’ experiences of online comparisons mirrored those reported in the existing literature from general population studies. However, differences in experiences were seen in two regards. First, the aforementioned symbolic metrics of connection meant that musicians’ successes and failures were directly comparable and public in visible hierarchies of status. While such metrics are relevant to the general population, they are particularly pertinent to the life of the musician, in that they are linked to (and present for) their personal *and* professional identities, and the mechanisms by which they felt their careers would be judged by others, i.e., those with whom they were seeking to build connections. Thus, Participant 2 described a “fear of the amount of ‘likes’ I get, or the amount of interaction, or whatever, being evidence for my career failing … It exists publicly and then you can think that everyone is thinking that.” In response, he had made the ‘likes’ on his Instagram private, but his management had asked him to make them public again because the visibility of the metrics would, they believed, “drive engagement,” again speaking to a sense of being tethered to the technology to some extent. What Participant 1 referred to as ‘the signal’—the metrics of the quantification of career musicianship—and by extension one of the fundamental rationales for SM use for musicians, was therefore itself a source of anxiety.

Secondly, while many interviewees fully recognised that the online profiles and posts they were seeing were, to some extent, an idealised and heavily curated representation, they still spoke of the impact these had on their wellbeing. In addition, interviewees spoke of actively curating their *own* online identities, but this not preventing upward comparison: “just because you’re aware of it being a marketing tool, that [doesn’t mean] it doesn’t affect you emotionally on a personal level, and I think that’s also difficult.” (P5). In other words, even though participants often had a high degree of digital literacy, this did not stop them being negatively impacted by what they were seeing online or playing a part in the process; indeed, often *because* they understood the landscape of the music industry so well, and recognised “the huge gulf between what a lot of musicians are saying online and how it really is in real life” (P6), this is some respects made the impact on their wellbeing worse. One of the highest status musicians we spoke to phrased this as: “there is a two-facedness and fakeness on SM [with musicians]… It’s just continuously, ‘everything’s amazing’. Which everyone fucking does. It’s a lie” (P6).

### 3.4 Making ‘content’, drawing boundaries

Such issues around self-presentation also raised questions about what to share and what not to share, as well as how and when to draw boundaries between online and offline, public and private, professional and personal selves. For some, this resulted in the establishment of clearly demarcated and hard boundaries, for others a complete lack of boundaries, with most landing somewhere in between. However, all participants shared a sense that what was being shared online was rarely the ‘full story’. Participant 5 referred to this as “curated authenticity.” These curations were often done with specific career-related motivations in mind: “Everything I post is always a mindset of like: is this going to sell me? So, it’s kind of like a sales pitch really in videos and photos” (P4).

With respect to what to share or not share online, while participants talked about sharing their music on SM, more often than not there was also an understanding that participants were sharing (and expected to share) something more of themselves through the creation of ‘content’ (see Negus, 2019): educational videos, ‘how-to’ guides, ‘behind the scenes’ footage, Q&As, a ‘day in the life’, footage in the studio recording a song, photographs, memes, videos of things they found inspirational, and more: “SM [has] become a short form content platform and you’re competing against content makers, right? People who make entertainment. Yeah. And you have to put music into that system” (P1). Participant 9 framed this as: “I’m sort of, you know, just thinking about other ways that I can post about the music without making it a sales pitch.”

While some participants embraced this as part of their creative process and an opportunity to share their musical journey, as well as conceptualising SM as a tool which could facilitate connectivity [“I actually love posting images, you know, making images of landscapes and this sort of urban landscape…. So I will post a lot of images like that and that’s not directly selling anything but that’s just part of who I am” (P6)], the majority appeared to, at least in part, resent being reconstituted as ‘content creators’. For example: “You’re expecting me to be an influencer or something, you know? You’re expecting me to be a YouTube personality; a character capable of turning my life into content and fronting it like a fucking Good Morning TV presenter” (P2), or “I absolutely hate it…This ‘having to be a video editor’. It’s constant. It’s just this pressure to do that much content” (P7).

Perhaps the clearest manifestation of the centralisation of content creation in the participants’ lives was that during our interview, Participant 1 attached his phone to a microphone stand and began filming his face as he answered as he thought his responses could be edited together to make engaging content for him to share on Instagram.

This pervasive and all-encompassing content-creation [“Every day, post every day, post every day, post every day, edit, edit, edit, what’s a good idea, what’s the content?” (P1)] was so central to some participants’ lives that they described their quest to produce engaging content as impacting their real-world choices and decisions; something Prey and Lee (2024) in their work on Korean independent musicians call “creator creep,” i.e., when the practices of content creation ‘creep’ into all areas of musicians’ lives. For example, Participant 2 told us: “I’m going on holiday and, you know, the conversation is like, ‘oh we could get good content there’… It’s pretty grotesque… It has now kind of co-opted everyone’s brains to just seeing their life and their experiences through some opportunity to create more content” (P2). For Participant 4, being a content creator even impacted her creative and artistic decisions; she suggested that she would sometimes pick certain songs to play at a live show “with the thought process that ‘I’m going to get this reaction on video and then post it to my TikTok’.” Woods (2023) has referred to this as a ‘focus group’ approach to music-making where online trends come to determine creativity itself: as Participant 6 phrased this to us: “it used to be more like I was in the real-world making art and writing music, and now [that music] almost has to fit into social media.”

In this context of all-encompassing content creation, interviewees talked about needing to draw conceptual boundaries around various areas of their lives, principally their time, and their relationships with listeners. With reference to the latter, while developing deep relationships was understood as one of the primary rationales for SM usage, this could present challenges vis-à-vis drawing and maintaining boundaries. Participants 2 and 4 (both high-status musicians) both had experiences of stalkers, with the former even needing to involve the police. Participant 6 explained this as a need for “edited closeness.”

However, the clearest example of the impact of boundaries (or lack thereof) on wellbeing concerned time, and in particular, the time SM content production took up. This was not only in terms of ‘headspace’ and its negative impact on creativity and feelings of exhaustion, but also in terms of physical time spent away from loved ones. A number of participants, in fact, connected their sense of exhaustion with the fact that SM impacted other areas of their lives, from time they could spend making music, to time with friends and family. This was perhaps most clearly articulated by Participant 1 in an extended extract below:

“We work in the factory - the content factory - to create the content. We don’t make any money off that content. [Meta] take that content. We sign a contract. And they use that content to create data that they sell and then we pay them to be seen with either our time or our money… The time is spent not just making, but learning, and practising… Time is the biggest cost… Time is at the centre of everything… My main thing is being there for my girlfriend and being present for my children… But being present matters, and being present in the arts matters. Then you’ve got this balance of being present in these two worlds. How do you balance that presence?’ (P1).

### 3.5 Moderating factors

Finally, thematic coding revealed four factors that appeared to impact the relative strength or weakness of the effect of SM on participants’ mental health and wellbeing. These were: (i) career orientation and status, (ii) gender, (iii) race, and (iv) attitude towards SM.

With respect to career status, a less healthy relationship to SM was common among two groups. The first were those who had a more explicitly commercial orientation to their music, i.e., those who saw music as (or desired it to be) their primary career as opposed to something meaningful and important but which they did alongside other kinds of work. The second were those who worked in more commercial genres of music, e.g., pop, house, compared to participants who worked in slightly more niche genres, e.g., punk (although it is important to acknowledge only two participants fell into this latter category). Commercially-minded musicians tended to report greater anxiety over their metric visibility, be more aware of, and impacted by, practices of comparison, and under greater (perceived) pressure to produce content more regularly.

Relatedly, the status of the musician was also key. Artists with smaller followings on SM suggested they were less vulnerable to some of the worst excesses of SM use. However, while being a larger and more visible artist was seen as bringing more vulnerabilities, there was a perception that there was a level of status beyond which SM was no longer necessary, and where having achieved this status participants hoped to be able to step away from SM. Indeed, this was the level many participants hoped that they were on their way towards, though none felt they had reached. However, given that we interviewed several musicians who were already well-established, it was not at all clear when one would reach this digital Promised Land: “You go past a certain point, and then I do think that you have the privilege of not having to use [SM]. But like I’m not there” (P5).

Gender also impacted participants’ experiences on SM vis-à-vis their health and wellbeing, sometimes in contradictory ways. On the one hand many of the women we interviewed shared their experiences of misogyny and abuse online, which often tended to centre around questions of competency. Thus, these women’s experiences online reflect offline misogyny in the music industry (House of Commons, 2024) and indeed wider society. However, Participant 4 suggested this was amplified online: “I think [the misogyny] is louder online in terms of, like, they’re saying exactly what they’re thinking” (i.e., reflecting the disinhibition effect; Joinson, 2007). On the other hand, however, Participant 5 suggested that SM can be used to cultivate supportive female spaces, as well as spaces of Black and Queer solidarity, which had been a source of great comfort to her.

For non-White participants, race and ethnicity was another key variable influencing the ways in which SM impacted their wellbeing. As Participant 5 told us: “You just deal with *so* much stuff as a Black person that, like, if you complain about all the things that happen then you just wouldn’t have any energy… I’ve felt lonely in that space.” Likewise, one participant experienced the intersections of both race and geography, living as he did in a non-diverse part of the UK. On the one hand, this meant that he was vulnerable, experiencing appalling racism as he tried to build his music career: “I love electronic and music of African origin, and I want to merge the two of them together. Through that I’ve had people who I didn’t even know just saying like ‘oh you should kill yourself’… Even some people that I did know, that like I went to school with, someone was saying that I should get run over” (P12). On the other hand, as per the contradictory role of gender, SM also offered communities of solidarity, with Participant 12 also referring to ‘Black Twitter’: “The discussions that I had on there, they just sort of show me that there’s other people who are going through a lot of the same stuff… I learned a lot about myself…. because where I was going to school… there weren’t any Black people… It’s only through Twitter that I was finding a community… Even though I’m getting people saying stuff about how I look, I’m also getting people who are empowering me,” and “I guess with all those experiences, they made me more aware of myself as a Black person, just in my place in the world, and probably contributed to me wanting to push through more African music, but also I know that in this country I can never really feel like I’m fully accepted” (P12).

Finally, the impacts of SM also appeared to be moderated by participants’ attitudes towards SM. Some appeared to openly embrace it as a creative tool, an extension of their music artistry, and part of their lives and practice, e.g., P1, P4 and P11. While these participants still felt SM could impact on their mental health and wellbeing negatively, they seemed more at peace with the role it played in their lives. One participant who was noticeably less career-oriented than the others, viewed SM more as a source of fun (albeit one which could be an irritant also) e.g., P10. However, others seemed to fundamentally resent it as an imposition; an unwanted extra which was burdensome and took focus away from the music they wanted to make, e.g., P2 and P7. The majority seemed somewhere in between, where they pragmatically accepted it as an inescapable part of the contemporary music landscape, but were actively seeking to work on their relationship with SM and the impact it had on their wellbeing, e.g., P3, P5, P6, P8, P9, and P12.

## 4 Discussion

Each of the five first-order themes will be considered in turn below. We will draw on relevant literature as well as the TCBC (Tibber and Silver, 2022) to contextualise and make sense of the findings. In addition, we will reflect on the implications of the findings for Tibber and Silver’s (2022) SM-specific TCBC, with a focus on any points of divergence, i.e., where the conceptualisation needs to be updated to be of relevance to this population, before turning finally to the clinical implications of this study.

At the broadest level, our findings concerning musicians’ motivations for use are supportive of a large body of literature and theory, including the Uses & Gratifications theory (U&G) (Ruggiero, 2000), as well as the TCBC (which draws on U&G), in recognising that individuals have agency, turning to SM to seek gratification of certain needs (social and professional), and critically, making active attempts to try and manage their use, i.e., to harness its benefits and ameliorate its harms. For many participants, the desire for connection was a deep yearning to be seen, heard, and understood, relating to a (near) universal and fundamental need, we would argue, to feel connected (Ryan and Deci, 2017). Such authentic connection and validation were linked to positive mental health and wellbeing, a finding consistent with the TCBC, which proposes that the experience of connection/disconnection is one of the main determinants of whether the SM user encounters benefits/harms, respectively; see the *interpersonal connection behaviour framework* also (Clark et al., 2018). Thus, while musicians may turn to SM predominantly for career progression, it is likely that as with non-musicians, SM has the potential to impact on mental health through its capacity to both connect and disconnect.

However, with reference to *external* control, participants primarily expressed uncertainty with respect to ‘the algorithm’ and its influence over their career, which is increasingly built and cultivated online (e.g., Baym, 2012, 2015; Woods and Davis, 2024; Watson et al., 2023), and therefore uncertainty over their ability to build the very connections which they felt were so valuable. Drawing on the TCBC, some distress or anxiety is inevitable when gratifications sought (career development) are not obtained, and there was uncertainty as to what online behaviours would be likely to increase the likelihood of them being met. Uncertainty around the nature of the algorithm, for example, mirrors the uncertainty of music careers characterised by risk-taking (Musgrave et al., 2024). Furthermore, this speaks to a wider, core underlying precarity of the musicians’ position; what Vachet (2024, p.4), drawing on Laing (2010 [1960]) calls a lack of ontological security, i.e., an experience of precarity or precariousness which is “not limited to labour conditions under post-Fordist economies but extending to all aspects of life and becoming an essential element of contemporary forms of subjectivisation.” Thus, it is possible that some of this may be projected onto the technology itself, and the demands thereof. The TCBC also speaks to the ways in which SM platform features may exacerbate such feelings of uncertainty and precarity, and why engagement is maintained in the face of these. For example, notifications and status updates, which follow an intermittent reinforcement schedule, alongside near-constant social comparisons (more on this below), likely feed the promise that success is possible, and that the ‘gods of the algorithm’ might pick you at any moment, or that musicians might only be one piece of great content away from their lives changing for the better.

With respect to *internal* control, participants often felt they lacked agency in their engagement and, as per O’Reilly et al. (2018), regularly employed the language of addiction. Participants reported that they would—despite the best of intentions—find themselves passively scrolling online, and subsequently, feeling guilty about time they lost to other, more valued activities. Consistent with self-determination theory (Ryan and Deci, 2017), and the TCBC, this suggests there is something implicitly disconcerting or distressing about SM use when it results in a feeling of decreased autonomy. Second, platform design is relevant here also, i.e., the algorithmic pull towards continuous engagement, with features such as the infinite scroll and automatic replay minimising opportunities to pause and process information deeply (Marek, 2023), such that users may be, as per the title of their paper, ‘*carried away by the TikTok algorithm*’ (Schellewald, 2021). Within the TCBC, this is captured as a mindless, habitual, automatic mode of engagement, as opposed to a more mindful, intentional, and purposeful one.

With respect to the third theme, *the experience of comparison*, findings are consistent with a wealth of literature from the general population in suggesting that upward social comparisons can have a negative impact on wellbeing and mental health (see review by Verduyn et al., 2020). While potential benefits of comparisons were also seen, e.g., comparisons driving inspiration (as per Meier and Johnson, 2022) and ‘benign envy’ (Tibber et al., 2024), negative effects were more commonly reported. While such comparisons are prevalent offline, they may be exacerbated online by the features and affordances of online communication, and further, may be particularly relevant to musicians. Increasingly, SM communication channels are highly visual, public and quantifiable (Nesi et al., 2018), with social metrics such as ‘likes’ and ‘shares’ providing a tangible if reductionistic (and arguably unreliable) indicator of success and worth which can be directly and quantitatively compared. Given the close connection between music-making and a musician’s identity (Beech et al., 2016), and our participants’ acknowledgement that their SM use took place in the context of highly curated and idealised self-representations, this may make this occupational group particularly vulnerable to the potential negative impacts of online comparison.

The fourth theme—*making content, drawing boundaries*—related to participants’ broader experiences of what is expected of a musician on SM nowadays in terms of what ought to be shared and created. For some, the injunction to share a range of ‘content’ was seen as positive, and from the perspective of the TCBC, an opportunity to satisfy core needs relating to autonomy, relatedness and competence (e.g., to be creative, take control of their career, and connect with audiences in an authentic way). However, this could also be seen as an imposition and intrusion, placing greater demands on musicians’ personal lives and shaping participants’ artistic and creative decisions (arguably reducing autonomy), and ‘displacing’ (Hall and Liu, 2022) other valued activities, e.g., eating into time that might otherwise be spent with loved ones, i.e., reducing relatedness.

With reference to moderating factors, there was a greater tendency for female and minoritised (e.g., Black) musicians to encounter greater levels of misogyny/racism/abuse, trolling and hate, reflecting offline power imbalances and social inequalities, both in the music industry as well as in wider society (Fox et al., 2015; Scharff, 2015). However, in parallel, there was also a sense that online spaces could afford a sense of community, shared experience, and even empowerment, highlighting, as discussed in the TCBC, that technology is neither positive nor negative, but a tool that provides opportunities for harms and benefits, particularly in relation to experiences of connection and disconnection.

The emergence of status and career orientation was interesting, both in terms of what was self-reported, and what was projected on to others. There was a sense that being a relatively early career or low status musician may offer protection from some of the risks experienced by those in the limelight, as well as a belief (not necessarily manifested) that very high levels of success might confer some protection. This, again, speaks to the role of social comparisons, and idealisation of the other in the context of an online space that is characterised by heavily curated self-presentations (Rauh, 2024) i.e., a positivity bias (Schreurs and Vandenbosch, 2021). This is particularly emphasised in the context of a musical economy which, as participants reflected on, demands outward facing positivity (see Gross and Musgrave, 2020) and one that may be fuelled by certain features and affordances of the technology itself. For example, more and more SM platforms now include the ability to share highly visual (and personal) content (i.e., pictures and videos), which can be heavily edited and curated (for instance through easy-to-use filters) and widely shared to a large audience (e.g., Hong et al., 2020), who subsequently indicate their approval and disapproval through comments, but also, quantifiable social metrics such as ‘likes’ and ‘shares’, that can be taken as a measure of one’s worth or status.

That less commercially-focused participants, and those within less commercial genres, e.g., punk, experienced less distress in relation to their SM use echoes findings in the broader literature that have suggested career-oriented musicians (sometimes called professionals) experience worse mental health and wellbeing than those who pursue music for leisure, recreation or a hobby (sometimes called amateurs) (Bonde et al., 2018; Loveday et al., 2023; Musgrave, 2023a; Musgrave et al., 2025). This may reflect, to some extent, a greater connection to, or sustained grounding within, underlying values, and a focus on intrinsic motivations, e.g., self-expression and creativity that are intrinsically rewarding, as opposed to a focus on extrinsic motivations, e.g., seeking status and success as a pathway to satisfaction of some other need(s). Values-consistent behaviour (Bramwell and Richardson, 2018), and intrinsic motivations (Ryan and Deci, 2000), have been linked to more sustainable motivation and better mental health. However, in the context of a highly competitive and commercialised industry such values and motivations have been seen to be sites of ongoing negotiation and tension by musicians (Everts et al., 2021).

Finally, participants’ attitudes towards SM itself also shaped their experience of the technology, with individuals who were able to embrace SM as an extension of their creative process tending to fare better. We would suggest that this may too reflect a deeper connection to, and sustained grounding within, their underlying values and intrinsic motivations (e.g., using the technology in the service of their creativity), as well as potentially a more mindful pattern of engagement that is aware of their online self as just one part of themselves, i.e., seeing SM as a tool with multiple uses that can be appreciated for what it is, while recognising the potential benefits and harms that it brings.

### 4.1 Implications for the TCBC

As demonstrated above, most of the findings reported can be successfully accommodated within Tibber and Silver’s (2022) SM-specific TCBC. In line with the conceptualisation, the findings suggest that participants were more likely to access the benefits of SM and experience positive impacts vis-à-vis wellbeing and mental health when they led to a satisfaction of core needs, particularly those relating to autonomy, relatedness and competence. Further, the findings suggest that this was more likely when participants engaged with the technology in an intentional/mindful and values-consistent way, but simultaneously reinforce the challenges of maintaining such a healthy pattern of engagement in the context of technological features and affordances that drive social comparisons and attentional capture.

However, while the TCBC offers a useful framework to make sense of users’ experiences, the findings also point to potential adaptations to the conceptualisation, or at least shifts in foci and additional factors to consider if it is to be of broad relevance and, ultimately, clinical utility to this population, i.e., career musicians in popular music genres.

First, while the TCBC linked satisfaction and dissatisfaction of core needs relating to autonomy, relatedness and competence to costs and benefits of engagement, respectively, it emphasised *relatedness* over the other two constructs in this triad. While it is clear that relatedness remained central to the quality and outcomes of participants’ SM experiences in our sample, autonomy and/or a sense of agency (or lack thereof) also seemed *particularly* pertinent. This may, in part, reflect the reality of musicians’ (perceived) professional obligation to engage with SM, i.e., an *inability* to disengage (Tessler and Flynn, 2016), and relatedly, a sense of being at the mercy of ‘the algorithm’, which in truth (we would suggest) no one truly understands, thus further stripping the user of a sense of agency. Musicians also arguably have more to lose (as well as gain) from SM than the general population, since SM has a very real potential to launch an artist’s career (or equally, to lead to an artist being ‘cancelled’), potentially feeding into fears of agency and a lack thereof.

Second, our findings highlight the role of identity in shaping the users’ SM experience. While identity was discussed in the original conceptualisation of the TCBC (Tibber and Silver, 2022), this study highlights identity as a core issue that deserves more attention within the conceptualisation, particularly (in this sample) with respect to the role of ethnicity and gender (see also: Jones et al., 2025; Sutcliffe, 2024).

Finally, our findings also emphasise the importance of musicians managing boundaries when navigating their online world. Although the relationship between boundaries and mindful, values-consistent engagement are discussed above, no reference to boundaries was made in the original TCBC conceptualisation (Tibber and Silver, 2022). While our clinical experience suggests that managing boundaries and thoughtful reflection on different parts of self (online and offline, public and private) are relevant to the general population, the findings reported here (as well as our clinical experience of working with musicians) suggest that such issues may be *particularly* pertinent to the life of a musician. This is likely due to the aforementioned reality that musicians are professionally obligated to have an online presence, which leads to a further (personal/private) distinction, as well as the (typically) greater audience size to which the career musician is exposed (particularly at more advanced stages of their career), and relatedly, the greater level of scrutiny that is afforded to people in the public eye.

### 4.2 Clinical implications and future research

The clinical implications of our findings are reinforced by reports, peppered throughout the interviews, of how participants often took active attempts to resist the algorithmic pull towards mindless engagement, and employ strategies (psychological, behavioural and technological) to try and manage their use, both in terms of ameliorating the harms and accessing the benefits. Such attempts were often in line with our own experience of working clinically with professional musicians. On the basis of this, we suggest below a number of potential targets for intervention/change, which may be informative for musicians wanting to manage their own technology use, or those working with musicians—whether members of a professional team around a musician, e.g., managers, labels, socials teams, or clinicians working in the field—to help them cultivate a healthy relationship to SM. Further, given the utility of the TCBC in describing the experiences of the musicians we interviewed here, we would suggest that the TCBC provides a useful framework for formulating and planning such interventions, particularly if the adaptations described in the preceding section are taken into consideration.

First*¸* healthy engagement seems to be characterised by engagement that is aligned with the users’ values. Thus, participants who were not focused solely on commercial aspects of the work but were able to remain connected to underlying values, e.g., connection, self-expression, and creativity, tended to fare better in the online world, retaining a creative and playful relationship to the demands of a professional online presence, while not minimising or denying the negatives this also brought. Thus, clarification of values may be an invaluable way to support musicians to stay connected with aspects of the career that are implicitly meaningful and rewarding, and further, to notice when important aspects of their offline/non-professional life risk being displaced; more on this below (Hayes et al., 1999).

Second, healthy engagement seems to be characterised by purposeful and intentional, i.e., mindful, use. As has been suggested, when mindfully engaged, the SM user is less likely to be prone to attentional capture by algorithms that push for greater levels of engagement (and linked to passive use), more able to process information encountered in a deeper and less biased way (e.g., seeing through the positivity bias and identity curation of others’ profiles), and further, more able to stay connected with (and notice when they are deviating from) their values (Tibber and Silver, 2022). Crucially, given the centrality of control in participants’ wellbeing, mindful engagement is (for the reasons outlined above) more likely to be associated with greater perceived control; further, due to its connection to the associated notion of ‘acceptance’, mindful engagement is also more likely to be associated with a greater tolerance of uncertainty, for example in relation to the algorithm (Junça-Silva and Caetano, 2023). Supporting its role as a potential target for intervention, mindfulness has been shown to be amenable to training (Quaglia et al., 2016), and there is emerging evidence to suggest that it plays a protective role in the association between SM use and mental health (Jones, 2024). This is an area warranting further research in this population.

Third, healthy engagement seems to involve an awareness of, and clear demarcation of, one’s boundaries, e.g., between the online and offline self, and between personal and professional identities. This is not intended to be prescriptive with respect to where the individual places these boundaries, how much these identities overlap, or what is shared or kept private (which will understandably differ greatly between individuals). However, from our experience, and emerging from the findings reported here, we see that individuals who are aware of, and able to reflect upon, such boundaries tend to be more resistant to negative online experiences, more tolerant of uncertainty, and further, able to retain a greater and more stable sense of self, even in the face of challenges to identity and career that are experienced (both online and offline). Clearer demarcation of boundaries (e.g., personal/professional) will also increase the likelihood (described above) that the musician will remain connected to other valued activities such as connecting with family and friends. In terms of working with such boundaries, strengthening skills related to mindfulness and reflective capacity, as well as clarification of one’s values, are likely to aid in this process.

Finally, given the seeming centrality of (upward) social comparisons in driving some of the negative effects of SM, both in musicians (as described here), as well as in the general population, it is clear these must also be addressed. However, this is complex, since musicians often draw inspiration from other artists online and are actively encouraged to track their online performance (for instance their number of followers and ‘streams’), such that online comparisons are to some extent an inevitable part of a modern musician’s life. From our clinical experience, however, we would suggest that there are a number of ways to mitigate the negative effects, and potentially, harness the benefits of online social comparisons. These include: (i) increasing awareness of the online positivity bias and tendency for online identity curation (Hogan, 2010); (ii) as per above, cultivating mindful/intentional values-based engagement so that there is less chance of being pulled towards passive ‘doomscrolling’, which has itself been linked to social comparisons and feelings of envy (Verduyn et al., 2015), presumably because when one is not actively engaging with one’s interests online one is likely to spend more time passively scrolling others’ idealised and heavily curated feeds; and (iii) cultivating a healthy, broad and flexible sense of self (Godbee and Kangas, 2020), including through clearer demarcation of online/offline, public/private, personal/professional parts of self, so that challenges to one part of one’s identity (e.g., online/professional) are not perceived as a threat to one’s entirety. With respect to the teams around the musician, e.g., managers, labels, and socials teams, we would suggest that when directing musicians to other artist’s posts, e.g., to highlight potential directions for development, they should be very specific as to precisely which aspects of the content the musician should attend to; otherwise, in our experience, the artist may make general comparisons with the ‘target’ or comparisons on unintended dimensions, with potential negative consequences to their confidence.

With respect to future research, we think there is a need for both further foundational research as well as more applied, clinical research. With respect to the former, there is a need for further qualitative and quantitative studies to unpick the mechanisms underlying the potential harms and benefits of SM use among musicians, as well as explore in greater depth moderating factors and individual differences, e.g., race, ethnicity, gender, sexuality, neurodivergence, socioeconomic status, genre-specific differences, geography, and musical territories that influence these processes. With respect to clinical work, it is our hope that such research will feed into the development and testing of targeted resources and interventions to help musicians (and others in the creative industries and public eye) to thrive in the online world (see Musgrave et al., 2023).

## 5 Conclusion

By bringing together an interdisciplinary team of scholars to empirically explore the relationship between musicians’ SM use and wellbeing, and exploring this through the lens of a pre-existing theoretical framework (the TCBC), our findings highlight what Nickell (2020) described (in their study of independent musicians in Beirut) as the ‘*promises and pitfalls*’ of SM. Participants’ reflections emphasised both the potential benefits of SM—opportunities for social connection, self-expression, networking, career building and inspiration—as well as the potential harms—experiences of social disconnection, social comparisons, uncertainty, stigma, trolling, abuse, and a pressure to share more and more, with the risk that other valued activities, including in the offline world, are displaced. The findings also point to how features and affordances of the technology may pull the user more towards automatic and habitual engagement, predisposing them to more unhelpful patterns of use and outcomes unless these are actively resisted and overcome (as some did).

While many of the risks and benefits described in this paper are common to the general (i.e., non-musician) user, we would argue that the stakes may be even higher for career musicians. We suggest this given our participants’ sense of being tethered to the technology to survive in an increasingly digital and technology-dominated industry, and note that this is likewise occurring within a cultural context that frequently suggests musicians are more ‘in control’ of their careers than ever before as a result of new technological developments (Dumbreck and McPherson, 2016; Gross and Musgrave, 2020), despite suggestions (evident in our interviews) to the contrary. This seems to be manifesting as an emotionally challenging mismatch. Likewise, musicians may be additionally at risk given the role of social comparisons involving such tangible, public social metrics (which for musicians are central to their sense of personal *and* professional identities), the role of an audience and industry that demands greater and greater direct-access, as well as the challenges of balancing and placing boundaries between online/offline, public/private, personal/professional selves in this context. Taken together, we hope that the clinical recommendations made in this paper offer some strategies for musicians and/or the teams they work with in seeking to harness the benefits that can be derived from SM, while seeking to mitigate some of the most harmful tendencies.

The findings also highlight the potential value of the TCBC (Tibber and Silver, 2022) in making sense of these phenomena, as well as possible limitations therein, e.g., a need for greater understanding of the role of identity in shaping online experiences, and the role that one’s profession plays, e.g., in shaping motivations for engagement, as well as benefits and costs thereof. Finally, the study also points to targets for intervention or self-development in managing a musicians’ online life, most notably, the cultivation of mindful, intentional and purposeful engagement that is aligned with one’s values, and reflective of (and attuned to the needs of) different parts of the self.

Finally, it is important to acknowledge the limitations of this study. Firstly, while the sample achieved a good level of diversity on various metrics, it is still only a modest sample which acts as a first step towards exploring the complex ways in which SM impacts musicians’ wellbeing and cannot claim to be generalisable to all musicians. By providing the interview proforma we hope this might offer possibilities for other researchers to explore these questions with larger and even more diverse datasets. Secondly, as per much of the literature on musicians’ health and wellbeing, the study arguably contributes towards a bias focused on understanding musical careers in the Global North, in this instance the United Kingdom. This field of study would be enriched by exploring these questions in other music territories around the world to see what differences and commonalities might be observed. Thirdly, the qualitative understandings of mental health and wellbeing in this study would be complimented well by both quantitative work in this population and studies which adopt more precise definitions of levels of anxiety and depression and how these might be impacted by SM process and/or change over time. Fourthly, the focus of this study has been musicians in genres of popular music, and therefore does not speak to the challenges faced by *classical* musicians online, which may be distinct from those revealed herein. Finally, this study has provided a snapshot in time concerning musicians’ understandings of the links between SM and their mental health and wellbeing, but longitudinal studies over the course of musicians’ careers would also be a welcome addition to this literature.

## Data Availability

The datasets presented in this article are not readily available because ethics was not granted to share interview transcripts. Requests to access the datasets should be directed to George Musgrave, g.musgrave@gold.ac.uk.

## Footnotes

^1^The term ‘social media’ (SM) refers to online platforms - of which ‘social networking sites’ (SNS), e.g., Facebook, Twitter, Instagram, Tik Tok, etc., are a subset - that enable users to create uniquely identifiable profiles, connect with others, and interact through sharing pictures, comments, and reactions to content.

^2^A full interview proforma with each of these sections detailing the questions asked to all participants can be found in Appendix 1.

## Author’s note

Our title draws inspiration from Gill and Pratt’s (2008) ‘In the social factory’ paper on precarious cultural work, and was a quote from a participant.

## Appendix 1: Interview proforma

**General Use:**

- Which social media platforms do you use? *Professional/personal?*
- How much time do you spend on social media/each of these platforms a week? *Professional/personal?*
- Do you have separate professional and personal accounts? If so, why?

**Motivations/Gratifications Sought:**

- What do you use social media for/what do you hope to get from it? *Professional/personal?*
- Has this changed over the course of your career and/or level of success? Have expectations of you changed? If so, how?
- How important – and in what ways - do you think social media is to your career and/or musicians’ careers more generally?

**Technology Features and Affordances:**

- Do you use different platforms and apps for different purposes? How so? *Professional/personal?*
- Are there particular functionalities or features of particular platforms or apps that you find particularly helpful or unhelpful? *Professional/personal?*

**ONLINE BEHAVIOURS**

- What do you tend to do online? *Professional/personal?*
- How active are you online (e.g. passive “lurking”/“scrolling” vs. active posting/content creation)? *Professional/personal?*

**Self/Identity:**

- What parts of your self do you share/not share online? *Professional/personal?*
- How are your online and offline personas different/similar? *Professional/personal?*

**Others/Fans and Audience:**

- What are the main reasons you think your fans engage with you on social media?
- Do you think there are particular types of fans that are drawn more strongly to engage with artists on social media?
- What do you think constitutes “unhealthy” social media engagement on the part of a fan?
- Have you had any negative social media interactions with fans or others? If so, what were/are your coping strategies for these?

**Mode of Engagement:**

- When you engage on social media, to what extent do you think you do it purposefully, i.e. with a clear intention of what you want to get from it?
- When you engage on social media, to what extent do you feel in control, for example of how much time you spend on it.

**Consequences/Gratifications Obtained**/**Costs and Benefits:**

- What are the benefits to you, do you feel, of maintaining a presence on social media? *Professional/personal.*
- What are the drawbacks or costs, to you, do you feel, of maintaining a presence on social media? *Professional/personal?*
- Do the ways in which you use and experience social media affect your mood and mental heath?
- Do the ways in which you use and experience social media affect what you do offline?
- Do you think there are particularly ways of engaging or reasons for engaging that are associated more with benefits?
- Do you think there are particularly ways of engaging or reasons for engaging that are associated more with drawbacks or costs?

**Changes:**

- If you could change anything about the way you use – or are expected to use - social media, what would it be?
- If you could change anything about social media in general, what would it be?
- What do you think would happen if you stepped back completely from social media for, say, three months?

**Older Musicians:** How was your career different before social media?

**Younger Musicians:** How do you think your career would be/would have been different without social media?
